# Online Assessment of Cognitive Functioning: A Reliable Alternative to Laboratory Testing?

**DOI:** 10.1093/geroni/igaa057.1917

**Published:** 2020-12-16

**Authors:** Maximilian Haas, Sascha Zuber, David Framorando, Elissa El Khawli, Susanne Scheibe, Matthias Kliegel

**Affiliations:** 1 University of Geneva, Geneva, Switzerland; 2 University of Geneva, Geneva, Geneve, Switzerland; 3 University of Queensland, St Lucia, Queensland, Australia; 4 University of Groningen, Groningen, Groningen, Netherlands

## Abstract

As the population ages, risks for cognitive decline threaten independence and quality of life for older adults. Classically, psychological assessment tools to evaluate cognitive functioning are administered in face-to-face laboratory sessions, which is time- and resource-consuming. With the aim of reducing such costs, the present study set out to develop and validate two new online tools, allowing a rapid assessment of general cognitive abilities and of prospective memory. We collected data from 250 participants equally spread across the adult lifespan (aged 18 – 86). Results suggest that performance assessed via these newly developed online tools is comparable to performance in face-to-face laboratory settings. Our findings thereby indicate that these online tools can reliably measure cognitive functioning across the lifespan at a reduced cost, which may help detect individuals at risk of developing age-related cognitive disorders.

